# Inner retinal thickness and optic disc measurements in obese children and adolescents

**DOI:** 10.5935/0004-2749.20200047

**Published:** 2024-02-11

**Authors:** Evre Pekel, Selda Ayça Altıncık, Gökhan Pekel

**Affiliations:** 1 Eye Clinic, Denizli State Hospital, Denizli, Turkey; 2 Pediatric Endocrinology, Pamukkale University, Denizli, Turkey; 3 Ophthalmology Department, Pamukkale University, Denizli, Turkey

**Keywords:** Retinal ganglion cells, Optic disc, Nerve fibers, Pediatric obesity, Adolescent, Body mass index, Células ganglionares da retina, Disco óptico, Fibras nervosas, Obesidade pediátrica, Adolescente, Índice de massa corporal

## Abstract

**Purpose:**

This study aimed to evaluate optic nerve head parameters and inner retinal layer thicknesses in obese children and adolescents.

**Methods:**

Forty-one eyes of 41 pediatric obese participants and 41 eyes of 41 ageand sex-matched healthy controls were included in this study. Body mass index was calculated, based on sex and age, using body weight and height measurements. Blood lipid values (i.e., cholesterol, low-density lipoprotein, high-density lipoprotein, and triglyceride) were measured in obese participants. Optical coherence tomography was used to examine optic nerve head parameters, including rim area, disc area, cup-to-disc ratio, and cup volume, as well as the thicknesses of retinal nerve fiber layers and macular ganglion cell-inner plexiform layers.

**Results:**

Optic disc parameters were similar in obese and healthy children (p>0.05). The percentage of binocular retinal nerve fiber layer thickness symmetry was significantly different between obese and control groups (p=0.003). Compared to the control group, participants in the obese group exhibited thinner retinal nerve fiber layers in the superior quadrants (p=0.04) and thinner ganglion cell-inner plexiform layers in the superior-temporal sectors (p=0.04). There were no statistically significant correlations between the ocular parameters and lipid blood test values assessed in this study (p>0.05). Body mass index was significantly negatively correlated with the mean retinal nerve fiber layer thickness (r=-0.33, p=0.03) in the obese group. There was no significant correlation between intraocular pressure and body mass index (r=0.05, p=0.74).

**Conclusion:**

Compared to healthy children, obese children had greater binocular retinal nerve fiber layer thickness asymmetry and thinner retinal nerve fiber and ganglion cell-inner plexiform layers in several sectors. Blood lipid levels were not associated with retinal thickness or optic disc parameters in obese children.

## Abstract (pt)

**Objetivo:**

O objetivo deste estudo foi avaliar os parâmetros da cabeça do nervo óptico e a espessura da camada interna da retina em crianças e adolescentes obesos.

**Métodos:**

Quarenta e um olhos de 41 participantes pediátricos obesos e 41 olhos de 41 controles saudáveis pareados por idade e sexo foram incluídos neste estudo. O índice de massa corporal foi calculado com base no sexo e na idade, utilizando medidas de peso e estatura corporal. Os valores de lipídios no sangue (colesterol, lipoproteína de baixa e alta densidade e triglicérides) foram medidos nos participantes obesos. A tomografia de coerência óptica foi usada para examinar os parâmetros da cabeça do nervo óptico, incluindo a área da borda, área do disco, razão escavação/disco, volume da escavação, espessura s camadas de fibra nervosa da retina e as camadas plexiformes internas das células ganglionares da mácula.

**Resultados:**

Os parâmetros do disco óptico foram semelhantes em crianças obesas e saudáveis (p>0,05). A porcentagem da simetria da espessura da camada de fibras nervosas da retina binocular foi significativamente diferente entre os grupos obesos e controle (p=0,003). Comparados ao grupo controle, os participantes do grupo obeso exibiram camadas mais finas de fibras nervosas da retina nos quadrantes superiores (p=0,04) e camadas plexiformes mais finas da célula ganglionar interna nos setores temporal superior (p=0,04). Não houve correlação significante entre os parâmetros oculares e os valores dos exames de sangue lipídico avaliados neste estudo (p>0,05). O índice de massa corporal foi significativamente correlacionado negativamente com a espessura média da camada de fibras nervosas da retina (r=-0,33, p=0,03) no grupo obeso. Não houve correlação significativa entre a pressão intraocular e o índice de massa corporal (r=0,05, p=0,74).

**Conclusão:**

Comparadas às crianças saudáveis, as crianças obes as apresentaram maior assimetria binocular na espessura da camada de fibras nervosas da retina e fibras nervosas da retina mais finas e camadas plexiformes internas das células ganglionares em vários setores. Os níveis de lipídios no sangue não foram associados à espessura da retina ou aos parâmetros do disco óptico em crianças obesas.

## INTRODUCTION

Obesity is a public health problem among children and adolescents worldwide^(1,2)^. It is associated with several diseases, such as systemic hypertension and diabetes mellitus^(3)^. Possible mechanisms underlying the relationship of obesity and systemic disorders include oxidative stress, lipotoxicity, cardiometabolic dysregulation, vascular endothelial dysfunction, and chronic inflammation^(4-7)^. Many ocular diseases have the same abnormalities in their etiologies.

Ocular manifestations of pediatric obesity have been reported in several studies^(8-13)^. Notably, obesity may be associated with loss of retinal ganglion cells in children^(10)^. The retinal nerve fiber layer (RNFL) was found to be thinner in obese pediatric participants than in healthy controls^(11-13)^. In addition, several optic disc parameters, including disc area, cup volume, and cup-to-disc ratio, are reportedly affected in childhood obesity^(13)^.

In a previous study, we found that pediatric diabetes mellitus was associated with binocular RNFL thickness asymmetry, suggesting retinal degeneration^(14)^. Based on this outcome, we hypothesized that some metabolic syndrome components (i.e., excess body fat and high blood lipid levels) could be related to retinal and optic nerve head abnormalities.

In recent years, there has been an increase in the number of publications regarding obesity’s effects on the eye. However, the potential ocular effects of obesity are not yet well-established, and there is a lack of confirmatory data regarding the association between obesity and retinal ganglion cell axonal damage. This study’s purpose was to evaluate changes in the inner retinas and optic discs of obese children. We hypothesized that there might be some alterations in the thickness of the retinal nerve fibers and ganglion cell layers, as the inner retina is particularly vulnerable to the metabolic disorders and oxidative stress that are associated with obesity.

## METHODS

Forty-one children with obesity and 41 ageand sex-matched healthy controls were enrolled in this cross-sectional study, which was conducted at a tertiary setting (Pamukkale University Hospital) with the approval of the institutional ethical committee. The protocol adhered to the principles of the Declaration of Helsinki.

### Study population

All children and adolescents in the obese group had been diagnosed with obesity in a pediatric endocrinology clinic and subsequently referred to the eye clinic during the period from April 2016to December 2017. All patients consecutively referred to the eye clinic were recruited for the present study, if they fulfilled the inclusion criteria, to prevent potential bias due to sampling methods. The study and control populations were recruited concurrently. None of the participants had any ocular pathology or received medication before or during the study. Participants were excluded if they had a history of ocular surgery or disease (e.g., glaucoma, uveitis, amblyopia, retinal disorders, optic neuropathy, or trauma), if they had refractive error >2 diopters (D) spherical equivalent, or if they had a systemic disease that might affect optic disc and retina measurements (e.g., diabetes mellitus, thyroid diseases, hypertension, anemia, or heart problems). In addition, patients with intracranial hypertension, neurological diseases, and papilledema were excluded. All participants had good quality spectral domain optical coherence tomography (SD-OCT) images, with signal strength ≥7 (minimum: 1, maximum: 10), good centering, and uniform brightness. Systemic blood analysis values were recorded in the study group, including cholesterol, low-density lipoprotein, high-density lipoprotein, triglyceride, fasting glucose, and insulin levels. Body weight and height measurements were performed in accordance with standard protocols^(1,2)^. Body mass index (BMI) was calculated, based on sex and age, using body weight and height measurements. The standard deviation scores for body mass index (SDS-BMI) were between 2.0 and 3.0 in the study group, which indicated obesity. The control group had SDS-BMI values of -1.0-1.0. The homeostasis model assessment of insulin resistance (HOMA-IR) test scores were recorded for obese participants.

### Ocular examinations

**Figure 1:**
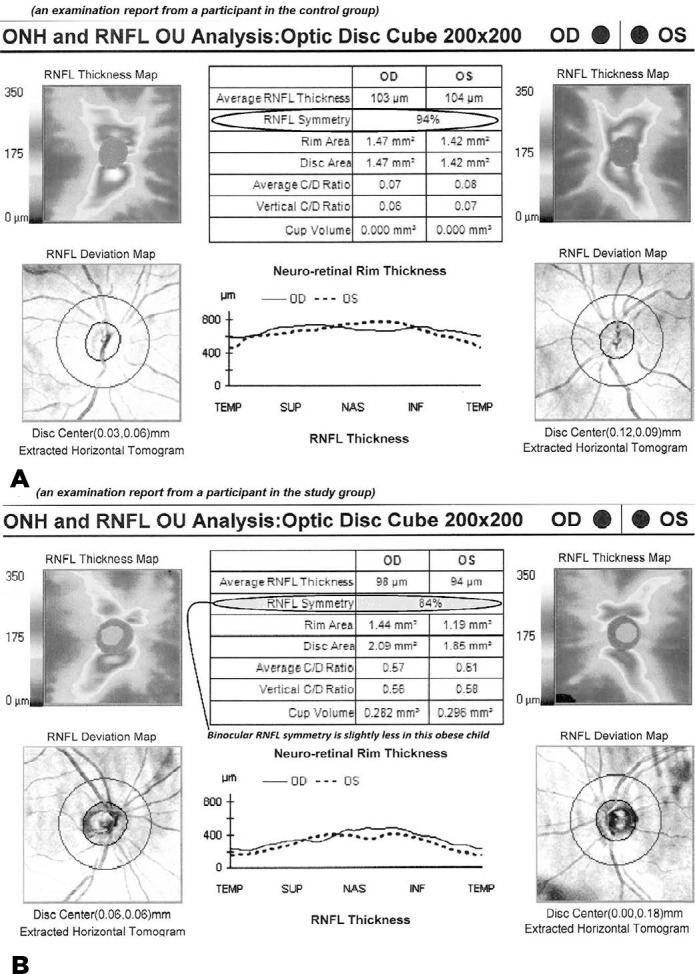
Combination of two OCT scans showing reduced RNFL symmetry in an obese child (B) compared to a control child (A).

All children and adolescents underwent ocular exa minations, including visual acuity assessments, ocular motility evaluations, slit-lamp biomicroscopy for ante rior segment examinations, air-puff tonometry for intraocular pressure (IOP) measurements, retinal evaluations, and SD-OCT measurements (Zeiss Cirrus HD 5000 model, Carl Zeiss Meditec, Dublin, CA, USA). The Cirrus HD-OCT 5000, which was used in the present study, has an A-scan velocity of 27000 scans/second with a 5-µm axial resolution and a scanning depth of 2 mm; the device uses a light wavelength of 840 nm and scans a 6 x 6-mm area of the macula. A circle with a diameter of 3.46 mm was automatically positioned around the optic disc to compute the mean and sectoral peripapillary RNFL thickness measurements. Optic disc parameters (i.e., disc area, rim area, cup volume, mean cup-to-disc ratio, and vertical cup-to-disc ratio), RNFL thickness, and macular GCL+IPL thickness measurements were recorded by the SD-OCT using an automatic segmentation protocol. Participants with potential SD-OCT segmentation errors were excluded from the study. RNFL measurements included mean thickness, quadrantal thickness, and binocular thickness symmetry analysis. Binocular peripapillary RNFL symmetry was defined as the percentage of RNFL thickness similarity between symmetrically opposed interocular peripapillary areas. Figure 1 shows a combination of two OCT scans, in which less RNFL symmetry is present in an obese child than in a control child. Mean, minimum, and 6-sectoral GCL+IPL thicknesses were analyzed for macular assessment.

### Statistical analysis

SPSS statistics software for Windows (version 17.0, SPSS Inc., Chicago, IL, USA) was used for analysis. All numerical data were expressed as mean ± standard deviation. P values <0.05 were considered statistically significant. To compare ocular parameters between obesity and control groups, independent samples t-tests were used. The Mann-Whitney U test was used to analyze the effect of sex on optic disc parameters in obese and non-obese participants. A Pearson correlation analysis was used to assess associations between ocular measurement and each of the following: systemic blood test values, BMI values, and HOMA-IR test values. The Shapiro-Wilk test was used to assess data normality. Only the right eyes of all participants were included in this analysis to eliminate selection bias.

## RESULTS

The mean ages of the patients in the obese and control groups were 11.5 ± 2.5 (range: 7-17) years and 11.9 ± 2.7 (range: 6-16) years (p=0.42), respectively. In both the obese and control groups, there were 12 male and 29 female children and adolescents. The mean refractive error (spherical equivalent) values of the obese and control groups were -0.04 ± 0.55 D and -0.15 ± 0.36 D, respectively (p=0.29). All participants had best-corrected visual acuity of at least 20/20 (logMAR 0.00). The mean IOP values of the obese and control groups were 17.1 ± 2.8 mmHg and 17.1 ± 2.6 mmHg, respectively (p=0.97). There was no significant correlation between IOP and BMI (r=0.05, p=0.74).

The mean peripapillary RNFL thicknesses in the obese and control groups were 97.2 ± 9.0 µm and 99.9 ± 9.6 µm (p=0.20), respectively. The RNFL thickness measurements in four quadrants are shown in table 1. There were no statistically significant differences in quadrantal RNFL thickness values between the obese and control groups (p>0.05) except for the superior quadrant (p=0.04). The percentage of binocular RNFL thickness symmetry was 86.2% ± 7.0% in the obese group, while it was 90.4% ± 5.1% in the control group (p=0.003).

**Table 1:** Quadrantal peripapillary RNFL thickness measurements in obese and control groups

	Obese group	Control group	p-value
Inferior quadrant (µ*m)*	128.2 ± 15.6	131.4 ± 18.4	0.40
Superior quadrant (µ*m)*	121.6 ± 15.2	128.9 ± 16.2	0.04
Nasal quadrant (µ*m)*	72.4 ± 11.6	69.8 ± 11.4	0.30
Temporal quadrant (µ*m)*	66.9 ± 10.8	69.2 ± 9.3	0.30

RNFL= retinal nerve fiber layer.

The optic nerve head measurements, taken by SD-OCT in the obese and control groups, are shown in table 2; those measurements were similar in both obese and control groups. The rim areas, disc areas, vertical and mean cup-to-disc ratios, and cup volumes were similar in boys and girls in the control group (p>0.05). In contrast, the mean cup-to-disc ratios (p=0.01), vertical cup-to-disc ratios (p=0.01), and cup volumes (p=0.006) were smaller in girls than in boys in the obese group.

**Table 2:** Optic disc parameters assessed by SD-OCT in obese and control groups

	Obese group	Control group	p-value
Rim area *(mm^2^)*	1.51 ± 0.27	1.57 ± 0.21	0.29
Disc area *(mm^2^)*	1.85 ± 0.39	1.85 ± 0.30	0.96
c/d mean	0.37 ± 0.18	0.34 ± 0.16	0.49
c/d vertical	0.35 ± 0.18	0.33 ± 0.16	0.63
Cup volume *(mm^3^)*	0.095 ± 0.099	0.070 ± 0.091	0.23

SD-OCT= spectral domain optical coherence tomography; c/d= cup-to-disc ratio.

The mean middle GCL+IPL thickness values in the obese and control groups were 83.5 ± 5.7 µm and 85.7 ± 5.8 µm, respectively (p=0.09). The mean minimum GCL+IPL thickness values in the obese and control groups were 80.4 ± 5.9 µm and 83.3 ± 6.2 µm, respectively (p=0.035). The sectoral macular GCL+IPL thickness values in the obese and control groups are shown in table 3. The sectoral macular GCL+IPL thickness values were similar between the groups (p>0.05) except for the superior-temporal sector (p=0.04).

**Table 3:** Mean sectoral macular GCL+IPL thickness values in obese and control groups

	Obese group	Control group	p-value
Inferior (µ*m)*	82.2 ± 6.3	84.4 ± 6.7	0.12
Inferior-nasal (µ*m)*	84.2 ± 6.5	86.4 ± 6.8	0.15
Inferior-temporal (µ*m)*	82.8 ± 6.1	85.0 ± 5.9	0.09
Superior (µ*m)*	84.1 ± 6.4	86.8 ± 6.4	0.06
Superior-nasal (µ*m)*	85.5 ± 6.2	87.1 ± 6.2	0.22
Superior-temporal (µ*m)*	81.4 ± 5.8	84.1 ± 5.9	0.04

GCL= ganglion cell layer; IPL= inner plexiform layer.

**Figure 2:**
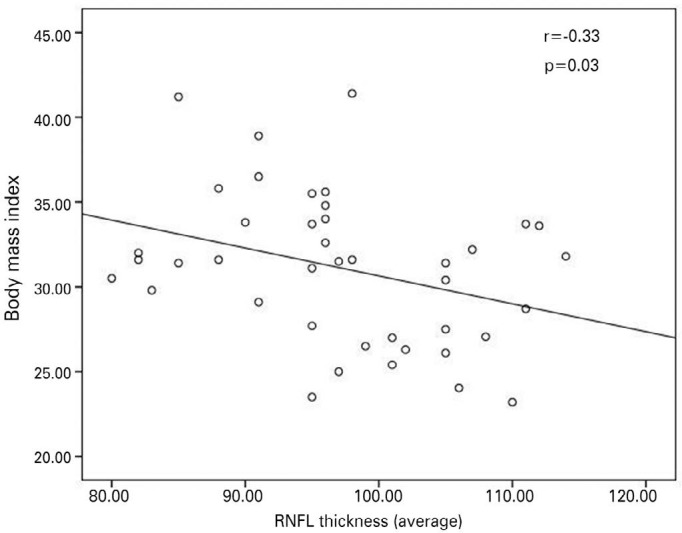
Body mass index (BMI) was negatively correlated with mean retinal nerve fiber layer (RNFL) thickness in obese children and adolescents.

The mean BMI value was 31.10 ± 4.45 (range: 23.20-41.40) kg/m^2^ and the mean SDS-BMI was 2.30 ± 0.22 in the obese group. Mean BMI was negatively correlated with mean RNFL thickness (Figure 2). BMI was not statistically significantly associated with binocular RNFL symmetry (r=-0.19, p=0.23), rim area (r=0.05, p=0.77), disc area (r=-0.07, p=0.65), mean cup-to-disc ratio (r=-0.08, p=0.64), cup volume (r=-0.11, p=0.51), mean GCL+IPL thickness (r=-0.28, p=0.08), or minimum GCL+IPL thickness (r=-0.23, p=0.16).

Systemic blood analysis values in obese children and adolescents were as follows: 160.5 ± 29.9 (range: 101-215) mg/dL for cholesterol, 110.5 ± 48.8 (range: 41-270) mg/dL for triglyceride, 44.1 ± 7.1 (range: 3167) mg/dL for high-density lipoprotein, and 94.0 ± 22.8 (range: 55-243) mg/dL for low-density lipoprotein. The mean fasting glucose value was 92.6 ± 6.9 mg/dL, while the mean blood insulin level was 17.1 ± 6.8 mIU/L. There were no statistically significant correlations between the ocular parameters and systemic blood analysis values in this study (p>0.05) except for a negative correlation between fasting insulin level and mean RNFL thickness (r=-0.33, p=0.04). The mean HOMA-IR value was 4.05 ± 1.68 in obese participants; this value was negatively correlated with mean RNFL thickness (r=-0.34, p=0.035), whereas it was strongly positively correlated with BMI (r=0.50, p=0.002).

## DISCUSSION

The present study associated pediatric obesity with a subset of inner retinal thickness changes. Notably, superior RNFL and superior-temporal GCL+IPL were thinner in obese children and adolescents than in healthy controls. The findings suggest that, in addition to the known complications of obesity, such as metabolic and cardiovascular disorders, the neurodegenerative effects of obesity should be assessed.

The present study adds some important new data to the literature regarding obesity. First, we found that the binocular RNFL thickness asymmetry was much greater in obese participants than in control participants. Mwanza et al. found that an interocular difference of mean peripapillary RNFL thickness might indicate early glaucomatous damage^(15)^. In addition, diabetic children had greater binocular RNFL thickness asymmetry than healthy controls^(14)^; to specifically focus on obesity as a metabolic syndrome, diabetic children were excluded from the present study, so that it only included obese children and adolescents. Second, we found that minimum and superior-temporal GCL+IPL thicknesses were lower in the obese group than in the control group. The GCL thickness is presumed to have a high correlation with visual field analysis and predicts future progression of several neuro-ophthalmic disorders^(16)^.

In a recent study, pediatric obesity was associated with thinner RNFL in all quadrants and thinner GCL+IPL in the inferior and superior-temporal macular areas^(12)^. Reduced RNFL thickness in obese children and adolescents has been reported in several other studies^(10,12)^. Demir et al. reported that GCL+IPL thickness was similar between obese and non-obese children^(11)^. Consistent with our present findings, Karti et al. found that BMI, insulin level, and HOMA insulin resistance were negatively correlated with RNFL thickness^(12)^. In our study, we did not find a relationship between blood lipid levels and retinal measurements; however, Karti et al. reported an association between triglyceride and RNFL thickness^(12)^. As in our results, superior RNFL is reportedly thinner in several degenerative disorders^(17,18)^. Conversely, with regard to reporting thinner inner retinal layers in some sectors in obese patients, the mean thickness values were within normal limits in the present study; therefore, those outcomes may not be clinically significant. In previous studies, a possible link was reported between obesity and neurodegeneration^(19,20)^. In the light of those data, we speculate that alterations in OCT parameters in the study group might have been related to neurodegenerative processes in obesity.

The present study showed no statistically significant differences between obese and non-obese participants in terms of optic disc parameters such as rim area, disc area, cup-to-disc ratio, and cup volume. In contrast to our results, Koca et al. reported that obese children had smaller disc areas, smaller cup volumes, and smaller cup-to-disc ratios^(13)^. They also noted that female participants had larger rim areas, smaller cup volumes, and smaller vertical cup-to-disc ratios than male participants in the obese group. Similarly, obese girls had smaller mean and vertical cup-to-disc ratios and smaller cup volume than obese boys in our study. Conversely, Elia et al. reported that sex did not affect optic disc parameters in Caucasian children^(21)^.

Several prior studies have investigated the relationship between obesity and IOP^(22-24)^. Akinci et al. rep orted that obesity was a risk factor for increased IOP in pediatric patients^(22)^. In a large, population-based study, obesity was found to be associated with increased IOP^(23)^. In addition, Yoshida et al. reported that high BMI was associated with high IOP^(24)^. In our study, IOP measurements were similar in both obese and non-obese children. As in our study, Albuquerque et al. did not find a significant correlation between BMI and IOP in children^(25)^.

The present study had several limitations. First, additional systemic blood tests related to obesity, including leptin and adiponectin, were not included in the analysis and should be performed in future studies for a more comprehensive assessment. Second, visual field tests were not included in the analysis and should be performed in future studies to determine whether clinical effects are present in relation to changes in RNFL and GCL thicknesses.

In conclusion, pediatric obesity may have detrimental effects on particular areas of RNFL and GCL. In particular, obese children showed greater binocular RNFL thickness asymmetry; BMI and insulin resistance were inversely correlated with mean RNFL thickness. These alterations suggest the presence of an early neurodegenerative process in obese children. Further longitudinal prospective studies are needed to confirm whether obesity has neurodegenerative effects on the retina.
